# Is Cantonese lexical tone information important for sentence recognition accuracy in quiet and in noise?

**DOI:** 10.1371/journal.pone.0276254

**Published:** 2022-10-25

**Authors:** Yuan Chen

**Affiliations:** Department of Special Education and Counselling, Integrated Center for Wellbeing (I-WELL), The Education University of Hong Kong, Taipo, New Territories, Hong Kong SAR, China; The Hong Kong Polytechnic University, HONG KONG

## Abstract

In Chinese languages, tones are used to express the lexical meaning of words. It is therefore important to analyze the role of lexical tone in Chinese sentence recognition accuracy. There is a lack of research on the role of Cantonese lexical tones in sentence recognition accuracy. Therefore, this study examined the contribution of lexical tone information to Cantonese sentence recognition accuracy and its cognitive correlates in adults with normal hearing (NH). A text-to-speech synthesis engine was used to synthesize Cantonese daily-use sentences with each word carrying an original or a flat lexical tone, which were then presented to 97 participants in quiet, in speech-shaped noise (SSN), and in two-talker babble (TTB) noise conditions. Both target sentences and noises were presented at 65 dB binaurally via insert headphones. It was found that listeners with NH can almost perfectly recognize a daily-use Cantonese sentence with mismatched lexical tone information in quiet, while their sentence recognition decreases substantially in noise. The same finding was reported for Mandarin, which has a relatively simple tonal system, suggesting that the current results may be applicable to other tonal languages. In addition, working memory (WM) was significantly related to decline in sentence recognition score in the TTB but not in the SSN, when the lexical tones were mismatched. This finding can be explained using the Ease of Language Understanding model and suggests that those with higher WM are less likely to be affected by the degraded lexical information for perceiving daily-use sentences in the TTB.

## Introduction

### The importance of lexical tones in sentence recognition accuracy

As a tonal language, Chinese differs from English in that tones are used in Chinese to express the lexical meaning of words. Therefore, it is important to examine the role of lexical tone in Chinese speech recognition. Most previous studies on this topic have focused on Mandarin lexical tones. There are four lexical tones in Mandarin according to pitch pattern: high level (Tone 1), rising (Tone 2), low dipping (Tone 3), and falling (Tone 4) [1]. Patel et al. [2] and Wang et al. [3] examined the importance of Mandarin lexical tones by flattening the fundamental frequency (F0) contours of Mandarin sentences. They found that the role of F0 information in Mandarin sentence recognition was important in noise but redundant in quiet for adults with normal hearing (NH).

However, although Patel et al. [2] and Wang et al. [3] altered the primary cues (i.e., F0 information) that provide lexical tone information, their participants may have been able to utilize secondary cues (e.g., amplitude envelope) for lexical tone perception, as amplitude envelope correlates with F0 contours [4, 5]. To address this issue, Chen et al. [6] used a text-to-speech (TTS) engine to flatten the lexical tone of each word in a sentence (i.e., Tone 1, high level). For example, the sentence “他穿了一件灰格子上衣 / He wears a gray plaid jacket / ta1 chuan1 le1 yi2 jian4 hui1 ge2 zi1 shang4 yi1/” was presented as “/ta1 chuan1 le1 yi1 jian1 hui1 ge1 zi1 shang1 yi1/.” This ensured that amplitude envelope did not carry the F0 contour of the original lexical tones. Their results showed that listeners with NH almost perfectly perceived the synthesized flat-tone sentences in quiet, but their sentence recognition declined substantially in speech-shaped noise (SSN) at 0 dB signal-to-noise ratio (SNR) (i.e., presentation level of the targeted sentence minus that of the noise) [6]. This was consistent with the results of Patel et al. [2] and Wang et al. [3], although different tone manipulation methods were used in these studies.

However, the above studies focused on Mandarin, and there is limited research examining the role of Cantonese lexical tones in sentence recognition accuracy. Mandarin has a relatively simple tonal system, where each lexical tone has a unique pitch shape. Cantonese, on the other hand, has one of the most complex tonal systems of all languages, where both pitch height and shape are used to contrast lexical items. It has three level tones (Tone 1 [T1, high level], Tone 3 [T3, mid-level], and Tone 6 [T6, low level]), two rising tones (Tone 2 [T2, high rising] and Tone 5 [T5, low rising]), and one falling tone (Tone 4 [T4, low falling]). These tones differ in pitch height, pitch contour shapes, or both. For example, T1, T3, and T6 are differentiated by pitch height. T4 and T5 differ by pitch contour shape (i.e., the direction of pitch change). T1 and T5 are contrasted by both pitch height and shape (see Wong & Chan [7] for a detailed description of the acoustic properties and pitch contours of Cantonese tones). Even native Cantonese-speaking adults may find it difficult to discriminate Cantonese lexical tones with different pitch levels but with similar shapes, such as T2–T5, T2–T6, and T4–T6 [7]. It is thus unclear whether the results of studies on Mandarin can be applied to Cantonese.

Additionally, only speech-spectrum-shaped noise (SSN) was included in Chen et al.’s study [6]. According to whether background noise is with linguistic context, masking could be referred to as informational (with linguistic context) and energetic (without linguistic context). SSN generates mainly energetic masking while two-talker babble (TTB) mainly generates informational masking [7, 8]. To further elucidate the role of lexical tones in speech understanding, it is important to evaluate how lexical tone processing affects sentence recognition in ecologically valid environments with multiple talkers. Therefore, the first aim of this study is to examine the contribution of lexical tone information in Cantonese sentence recognition accuracy in quiet and in noise. Both SSN and TTB were used to mask the sentences. In addition, unlike Chen et al. [6] who flattened the lexical tones of all words (Mandarin Tone 1, high level), in the current study, sentences were further categorized into Flat Tone (FT) 20%, FT 40%, FT 60%, FT 80%, and FT 100%, representing the manipulation of 20%, 40%, 60%, 80%, and 100% of words in a sentence, respectively. This made it possible to establish the relationship between sentence recognition accuracy and the amount of preserved lexical tone information.

### Role of working memory in perceiving sentences with mismatched lexical tone information in noise

According to the Ease of Language Understanding (ELU) model [9], working memory (WM) comes into play when there is any mismatch between the perceptual input and phonological/lexical representation stored in long-term memory [10]. WM plays a different role in *prediction* and *postdiction* under the ELU framework. The postdictive role is slow (on a scale of seconds), explicit, and thought to pertain post factum when a mismatch has already appeared. WM is deployed and used for making inferences and decisions to compensate for the mismatch. Conversely, the predictive role of WM is fast (on a scale of tenths and automatic), implicit, and associated with the ability to inhibit processing of irrelevant information [9, 11]. When the semantic contents of TTB are intelligible and compete with the target sentence, more WM resources for inhibiting the irrelevant information (*prediction* role of WM) may be exerted compared to the SSN [12]. WM plays a more important role in perceiving sentences with mismatched information in TTB compared to SSN [12].

However, the semantic context and predictability could mediate the effects of WM on perceiving sentences with mismatched lexical tone information in noise [11]. If sentence materials are high on contextual support and (or) predictability, the dependence on WM to compensate for the mismatch decreases [11]. This is because guesswork and inference-making are not needed to the same extent as for sentence materials with low lexical predictability and contextual information [11]. The sentences used in the current study are from the Cantonese version of the Hearing in Noise Test (CHINT), which are used for daily communication and are thus high on contextual support [11]. Furthermore, compared to vowels and consonants, there are fewer Cantonese lexical tones, making sentences with mismatched lexical tones higher on predictability. This may also decrease the reliance on WM for sentence recognition. Therefore, the second aim of the current study is to examine the role of WM in perceiving daily-use sentences with mismatched lexical tone information in two different types of noises (i.e., TTB and SSN).

## Methodology

### Participants

A total of 97 adults (25 males and 72 females) aged over 18 years and with NH were recruited from The Education University of Hong Kong. All participants (mean age = 21.59, standard deviation [SD] = 2.33) were native Cantonese speakers living in Hong Kong for at least 10 years. NH was defined as hearing thresholds better than 20 dB at octave frequencies from 250 to 8000 Hz bilaterally and the absence of outer- or middle-ear pathologies according to otoscopic examination, tympanometry, and medical history. In addition, all participants had normal or corrected-to-normal vision.

Considering the number of CHINT sentences and time required for testing, 63 participants attended study one to examine the contribution of lexical tones to sentence recognition in quiet and in the SSN and to compare the results of Mandarin from Chen et al. [6]. Another 34 participants attended study two to examine the relationship between WM and the ability to perceive sentences with mismatched lexical tones. The sample size was determined based on 80% power, a type I error of 0.05, and an effect size of 0.68 (Cohen’s d) (the difference in sentence recognition score declining from NT to FT 100% between Cantonese and Mandarin) for study one and an effect size of 0.42 for study two (the relationship between the decline in sentence recognition scores in TTB and WM). These effect sizes were from a preliminary study of 11 participants who met the above inclusion criteria.

### Sentence recognition

The speech material comprised sentences extracted from the CHINT corpus [13], containing 12 lists of 20 daily-use sentences, with each sentence consisting of 10 words. It has high inter-list reliability, suggesting that the lists are equivalent and can provide results consistent with each other [13]. The lexical tones of each CHINT sentence were manipulated to yield two test conditions using the NeoSpeech TTS software program: Normal Tone (NT) and Flat Tone (FT) conditions. In the NT condition, lexical tones within sentences were not altered, and the sentence quality was similar to that produced by Cantonese speakers in normal conversational style. In the FT condition, all manipulated lexical tones were changed to T1 (i.e., high level). Specifically, the characters in each CHINT sentence were first presented as a string of Jyutping (Cantonese phonetic symbols). The six lexical tones were represented by the digits 1 to 6. Changing the digits changed the tone of each word, as the stimuli were produced. For example, the stimulus “老闆經常去酒店食牛扒” (“The boss always eats steak at the hotel”) (/lou5 baan2 ging1 soeng4 heoi3 zau2 dim3 sik6 ngau4 paa2/) was changed to /lou1 baan1 ging1 soeng1 heoi1 zau1 dim1 sik1 ngau1 paa1/ in the FT 100% condition. All synthesized stimuli were produced at a sample rate of 16,000 Hz in a normal conversational speaking rate using a female voice with a mean F0 of 240 Hz. As mentioned earlier, according to how many words in a sentence were manipulated, sentences in the FT condition were further divided into FT 20%, FT 40%, FT 60%, FT 80%, and FT 100%, representing the manipulation of 20%, 40%, 60%, 80%, and 100% of words in a sentence, respectively. The words chosen to be manipulated were randomized. Our pilot study with 10 participants showed that words chosen did not significantly affect speech recognition as long as they were randomized in each sentence list (20 sentences) for each lexical tone manipulation condition. In spite of this, three sets of sentence lists were created, although only one set was randomly chosen to be administered to each participant. This practice was adopted to further counterbalance the number of critical/uncritical words, which may affect intelligibility to a greater/lesser extent, chosen to be manipulated across lexical tone manipulation conditions.

In addition, a continuous speech-spectrum-shaped noise, with the same long-term spectrum as the CHINT sentences, was used to corrupt the synthesized sentences at 0 dB SNR. A two-talker (one female and one male) babble noise from Wong et al. [7] was used as the TTB noise, which was recorded using 25 daily sentences selected from the CHINT, which include all the phonemes of Cantonese.

### Working memory

The Cantonese Reading Span Test (CRST) with dual-task design was used to measure the WM capacity. The CRST was developed following the design of Carroll et al. [14]. Validity and reliability have been assessed in young adults with NH [15].

A total of 54 sentences arranged in three blocks of either three, four, five, or six sentences were used. Participants were asked to read sentences presented phrase-wise on a computer screen aloud, at a rate of 1.2 seconds/word, with 0.1 seconds blank-screen intervals. Next, they were expected to judge orally whether the sentence is plausible (sensible) or absurd (senseless) within 1.75 seconds. After each block, participants were expected to recall either the first or the last phrase of each sentence. The final score represents the total number of phrases that an individual could recall. The entire procedure was carried out by a computer-based software.

### Procedures

The experiment was conducted in a sound-proof and sound-treated room, which met ANSI/ASA S3.1–1999 (R2013) standards for maximum permissible ambient noise levels for uncovered ears. The stimuli and noise were played at 65 dB (A) binaurally to participants through inset earphones (ER2, Etymotic Research). To familiarize the participants with the sentences and procedures before the test, each participant attended a training session and listened to two sentence lists in the NT and FT 100% conditions. For the actual test, each participant was evaluated under 12 test conditions and was instructed to repeat as many words as possible. The order of these conditions was randomized. The sentences were scored according to the number of words correctly repeated, and only exact matches in pronunciation were accepted (i.e., vowels, consonants, and lexical tones needed to be correct). The CRST was administered in the same room using a laptop with a 12.4-inch display screen, and a practice session was conducted before the actual test. Verbal instructions were repeated, and participants’ understanding of the instructions was checked prior to test administration. All experimental procedures were approved by the Human Research Ethics Committee of The Education University of Hong Kong. Written consent forms were obtained before the testing.

### Data analysis

Descriptive statistics were used to characterize the sentence recognition accuracy when the lexical tone was mismatched in quiet and in noise. Skewness and kurtosis were calculated to check the normality of the study variables. The criteria (skewness ≤ ± 3.0 and kurtosis ≤ ± 10.0) proposed by Weston and Gore [16] were used. The values of skewness and kurtosis for the study variables were all within acceptable ranges, indicating the data were close to normal distribution. Mixed model analysis of variance (ANOVA) tests were conducted to examine whether there was significant difference in the contribution of lexical tones to sentence recognition accuracy between Mandarin and Cantonese and between SSN and TTB. Pearson’s correlation analysis was used to examine whether those with better WM were less likely to be affected by the mismatched lexical tones. The Bayes factor (BF_10_) was used to quantify the support for the alternative hypothesis (H1: there were significant relationships between WM and the ability to perceive sentences with mismatched lexical tones) over the null hypothesis (H0: there were no significant relationships between WM and the ability to perceive sentences with mismatched lexical tones). A BF_10_ between 1 and 3, between 3 and 10, or greater than 10 is considered weak, moderate, or strong support for the H1 over H0, respectively. On the other hand, a BF_10_ between 1 and 1/3, between 1/3 and 1/10, or smaller than 1/10 represents weak, moderate, or strong support for the H0 over H1, respectively [17]. IBM SPSS Statistics for Windows, Version 24.0 was used to perform the above analysis.

## Results

### Sentence recognition accuracy in quiet and in SSN

**Fig 1:**
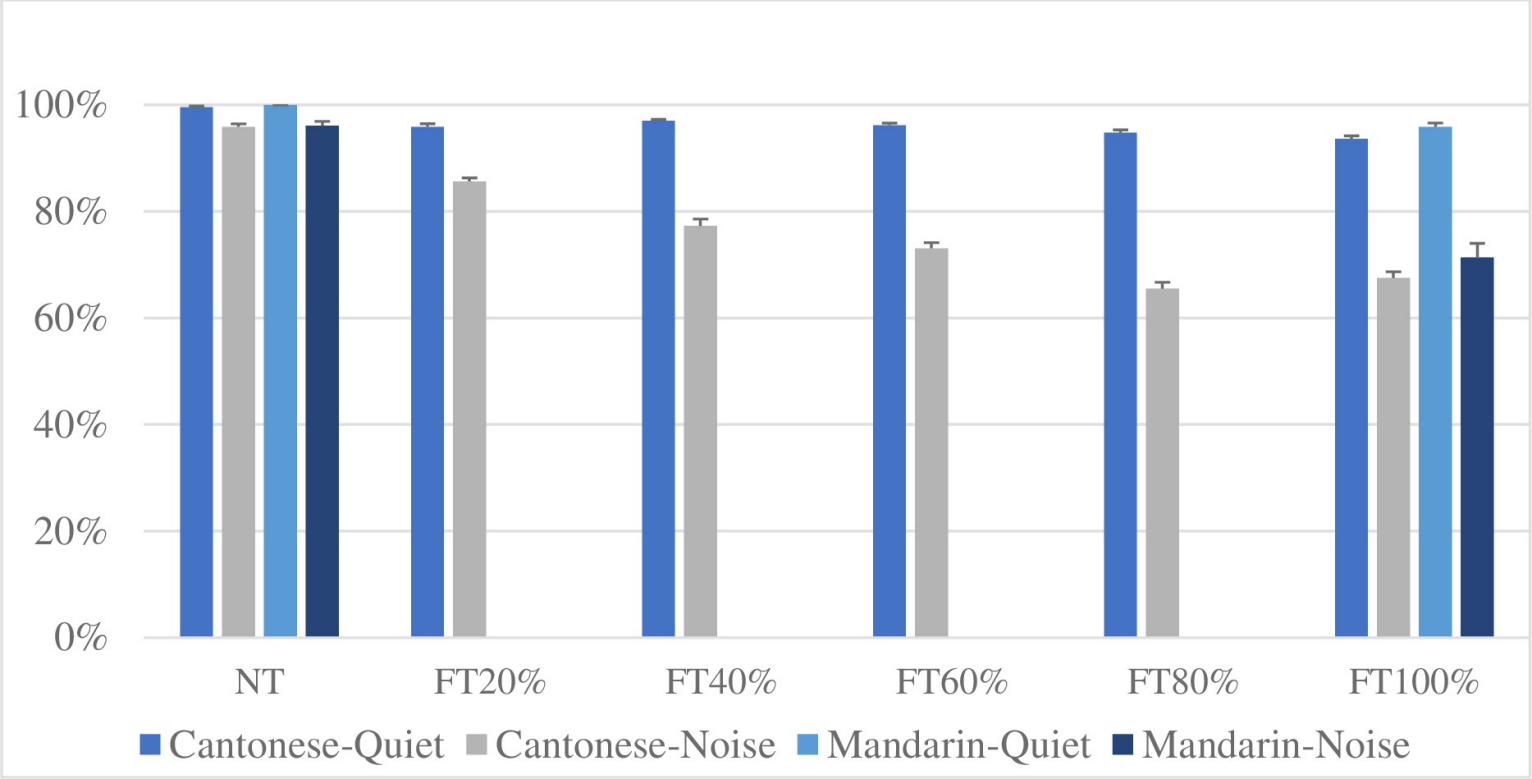
Mean Cantonese and Mandarin sentence recognition scores (percentage of words identified correctly) in quiet and at 0dB SNR with speech-shaped noise. The error bar denotes ±1 standard error of the mean. NT, FT 20%, FT 40%, FT 60%, FT 80%, and FT 100% represent 0%, 20%, 40%, 60%, 80%, and 100%, manipulation of words in a sentence, respectively. Mandarin sentence recognition scores were obtained from Chen et al. [6].

Fig 1 shows the mean sentence recognition scores declined with the loss of Cantonese and Mandarin lexical tone information. Mandarin sentence recognition scores were obtained from Chen et al. [6]. The mean Cantonese sentence recognition scores dropped by about 6 (SD = 0.04) and 28 (SD = 0.11) percentage points from NT to FT 100% conditions in quiet and SSN, respectively. According to Chen et al. (2014), there was a drop of 5 (SD = 0.02) percentage points and 25 (SD = 0.09) percentage points in the mean Mandarin sentence recognition score from NT to FT 100% in quiet and at 0 SNR with SSN, respectively. A mixed model ANOVA was conducted to evaluate the main effects of 1) listening conditions (quiet and noise), 2) languages (i.e., Mandarin and Cantonese), and 3) the interaction between listening conditions and languages on the decline of sentence recognition scores from NT to FT 100%. Results showed a significant effect of listening conditions (*F* (1, 72) = 142.50, *p* < 0.001), but the effects of languages (*F* (1, 72) = 1.62, *p* = 0.21) and interactions between listening conditions and languages (*F* (1, 72) = 0.41, *p* = 0.52) were not significant. This suggested that the importance of lexical tones for Cantonese sentence recognition accuracy in quiet and SSN is not significantly different from that for Mandarin.

In addition, one-way repeated ANOVA showed that there was a significant main effect of lexical tone manipulation conditions (NT, FT 20%, FT 40%, FT 60%, FT 80%, and FT 100%) in noise, *F* (5, 310) = 191.18, *p* < 0.001. Contrasts revealed that Cantonese sentence recognition score significantly declined with the decrease of the lexical tone information expect for the FT 80% and FT 100% test conditions. This suggested that although the lexical tone information was reduced in FT 100% compared to FT 80% test conditions, the sentence recognition scores did not significantly decline.

### Sentence recognition accuracy in TTB and SSN

**Fig 2:**
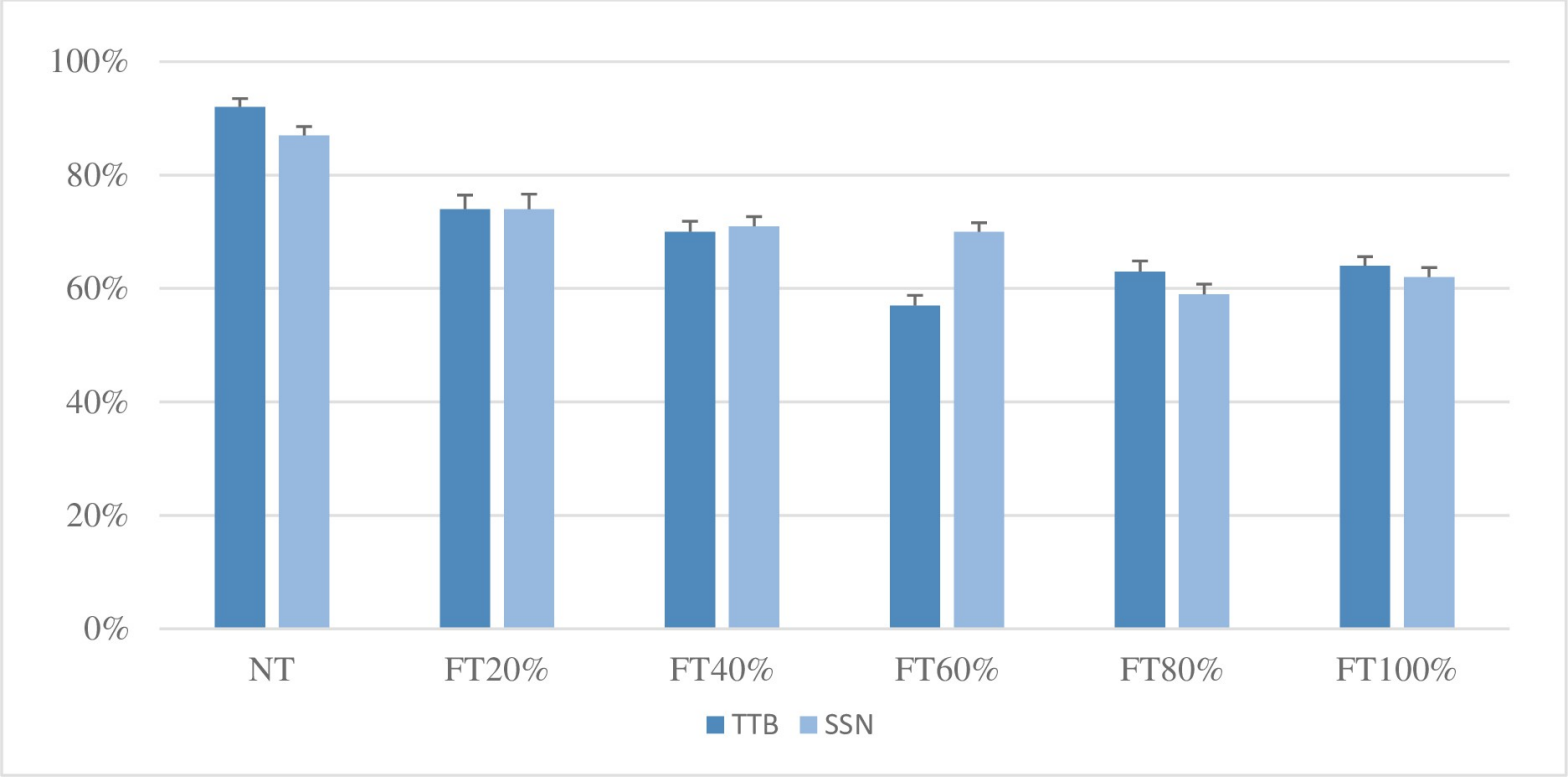
Mean Cantonese sentence recognition scores (percentage of words identified correctly) in speech-shaped noise (SSN) and in two-talker babble noise (TTB) conditions. NT, FT 20%, FT 40%, FT 60%, FT 80%, and FT 100% represent 0%, 20%, 40%, 60%, 80%, and 100% of words manipulated in a sentence, respectively.

Fig 2 shows mean Cantonese sentence recognition scores under SSN and TTB from Study 2. A mixed model ANOVA was conducted to evaluate the main effects of 1) lexical tone manipulation condition (NT, FT 20%, FT 40%, FT 60%, FT 80%, and FT 100%), 2) noise types (SSN and TTB), and 3) the interaction between lexical tone manipulation condition and noise types on the sentence recognition score obtained. Results showed a significant effect of lexical tone manipulation condition (*F* (5, 165) = 161.14, *p* < 0.001) and a significant interaction between lexical tone manipulation condition and noise types (*F* (5, 165) = 9.24, *p* < 0.001), but the effect of noise types (*F* (1, 33) = 0.09, *p* = 0.77) was not significant. Contrasts revealed that Cantonese speech recognition score in FT 60% was significantly lower than that in the other lexical tone manipulation conditions in the TTB. In addition, Cantonese speech recognition scores in FT 80% and FT 100% were significantly lower than those in other lexical tone manipulation conditions in the SSN. However, there were no significant differences in Cantonese speech recognition in FT 80% and FT 100% in the SSN, which was consistent with findings in study one.

These results suggested that the sentence recognition scores first declined and then increased with the decrease of the lexical tone information in TTB while they declined with the decrease of the lexical tone information and then plateaued at FT 80% and FT 100% in SSN (see Fig 2).

### Effects of working memory

The mean WM score was 29.24 (SD = 3.57, ranging from 23 to 39). As shown in Fig 2, the participants obtained the lowest sentence recognition score at the FT 60% test condition in the TTB and at the FT 80% test condition in the SSN. The maximum decline in sentence recognition score in TTB (i.e., the difference in mean speech recognition score between NT and FT 60%) and in SSN (i.e., the difference in mean speech recognition score between NT and FT 80%) was 35 percentage points (SD = 8.46) and 28 percentage points (SD = 13.74), respectively. Pearson correlation coefficients were computed, and results showed that WM was significantly related to maximum decline in sentence recognition score in TTB, *r* (34) = –0.39, *p =* 0.02, BF_10_ = 1.75, but not in SSN, *r* (34) = 0.01, *p =* 0.98, BF = 0.13.

## Discussion

In this study, the mean sentence recognition scores only dropped by about 6 percentage points from NT to FT 100% conditions in quiet, and about 28 percentage points from NT to FT 100% conditions at 0 dB SNR. This suggests that lexical tone information for Cantonese sentence recognition accuracy is relatively redundant in quiet but important in noise for adults with NH. This finding is consistent with those of Patel et al. [2] and Wang et al. [3], who examined the role of lexical tones in Mandarin sentence recognition using different lexical tone manipulation methods. They removed lexical tone information by flattening the F0 contours of Mandarin Chinese sentences. However, in the absence of explicit F0 information, amplitude envelope or duration can be used to perceive lexical tones. In this study, the TTS-synthesized sentences altered the F0 information, amplitude envelope, and duration, ensuring that participants could not use these primary and secondary cues for lexical tone perception.

However, although the TTS engine can synthesize sentences with artificially tonal contours, the generated prosody may not sound as natural as original sentences. Unnatural prosody may reduce the contrast between words, making it difficult to separate continuous speech into meaningful units [3, 18, 19], significantly decreasing sentence recognition in noise. Sentence recognition in quiet, however, is not substantially affected, as it achieves a high level (100% in the NT test condition). In addition, Feng, Xu, Zhou, Yang, and Yin [20] used sine-wave replicas of natural speech to examine the role of lexical tones in sentence recognition. They reported that sine-wave tone perception is largely impaired (to chance level), while the mean sine-wave sentence recognition is still accurate (i.e., 92% correct) in quiet. Sine-wave speech has more detrimental effects on acoustic cues, critical for lexical tone and sentence recognition (e.g., lack of harmonic structure and F0 variations), than the lexical tone manipulation method used in this study [6, 20, 21]. However, Feng et al. [20] found the same result as this study: limited functional load of lexical tones on sentence recognition accuracy in quiet. This suggests that the finding in quiet is robust and may not have been affected by unnatural prosody.

Furthermore, according to tone height and shape, there are three types of tonal languages. For example, many African languages use tone height, while Mandarin uses tone shape to contrast lexical tones. Cantonese and Thai have a more complex lexical tone system, wherein both tone height and shape are used to contrast lexical tones [7]. Despite these differences, the mean Cantonese sentence recognition scores found here are not significantly different from those of Mandarin as reported in Chen et al. [6]. The same lexical tone manipulation method and Hearing in Noise Test (HINT) sentences were used in Chen et al. [6]. However, the present results should be interpreted with caution because, although developed under the same paradigm, the equivalence of Cantonese and Mandarin HINT (e.g., the amount of contextual cues) have not yet been examined [13, 22]. Nevertheless, considering Cantonese has one of the most complex tonal systems, we speculate that the finding that lexical tones are critical for daily-use sentence recognition accuracy in noise but relatively redundant in quiet could be repeated with other types of tonal languages, although the specific decline in sentence recognition scores from NT to FT 100% may vary across languages. Further investigation of other tonal languages is thus warranted.

However, it is worth noting that the above speculation that lexical tone information is redundant for sentence recognition in quiet may not hold for sentences with limited context. Wang et al. [3] examined the role of lexical tone information and sentence context in Mandarin sentence recognition using normal and word-list sentences with and without F0 variations, which are primary cues for lexical tone perception, in quiet and in noise. Word-list sentences are formed using words pseudo-randomly selected from normal sentences. They are syntactically anomalous and semantically meaningless at the sentence level. Results showed that sentences with normal and flat F0 contours were more intelligible than their word-list sentence counterparts in quiet and noise. This suggests that the near-percent recognition in quiet may be attributed to the fact that the top-down information of sentence context could compensate the degraded lexical tone information. Therefore, lexical tone information may still be important for sentences with limited contextual information.

In addition, this study only examined the effect of reduced lexical tone on sentence recognition accuracy. It is possible that reduced lexical tone information may significantly affect reaction time and listening efforts. For example, an accent mark indicates lexical stress in Spanish words and is necessary for correct pronunciation of words. Marcet and Perea [23] found that the omission of the accent mark led to a cost in late (i.e., total reading time spent on the target word), but not early lexical processing in Spanish (i.e., fist-pass eye fixation durations). Furthermore, Zekveld et al. [24] found that listening efforts, measured using pupil dilation response during listening, changed across listening conditions and were related to interindividual differences in speech recognition. Therefore, although the mismatched lexical tone information has minimal effects on sentence recognition accuracy, it may significantly increase reaction time and listening efforts. Future studies are thus needed.

### The role of WM

This study demonstrated that WM was significantly related to maximum decline in sentence recognition score when lexical tones were mismatched in TTB, but not in SSN. The insignificant relationship between WM and maximum decline in sentence recognition score in SSN may be attributed to the contextual information of target sentences. Rudner et al. [25] reported that sentence recognition in SSN was dependent on WM only when the sentences had limited contextual cues (i.e., matrix-type materials). The WM dependency was not found for the HINT sentences, where context could be used to facilitate speech processing. The Cantonese version of HINT was used in the current study because it simulates everyday hearing situations, which is more ecologically valid and contains more contextual cues than the matrix tests. Therefore, the finding is consistent with the ELU model: context is assumed to aid listeners to unload WM because it could facilitate prediction and thus, no additional explicit processing is necessary for sentence recognition in noise [11]. This finding is also consistent with our previous study [26] conducted with Mandarin-speaking adults with hearing aids, where the Mandarin version of HINT and the same lexical tone manipulation methods were used; results showed that WM did not significantly affect sentence recognition in SSN among this population.

The significant and medium relationship between WM and the maximum decline in sentence recognition scores when lexical tones were mismatched in TTB may be attributed to the effects of WM on the low-level acoustic speech segregation (i.e., *prediction* based on the ELU model). More specifically, in the TTB, the semantic contents of the masker compete with the target sentence (i.e., information masking). This requires more WM capacity for inhibiting irrelevant information and overruling undesired responses [9, 12]. Additionally, the amplitude envelopes of the TTB being more similar to those of the target sentences than of the SSN may prevent the extraction of target speech from the background noise, requiring more WM capacity to grasp the target speech [12, 27]. Therefore, the effects of WM on the recognition of sentences of mismatched lexical tone information may be attributed to the modulation effects of WM on the acoustic speech segregation in TTB.

In addition, the significant relationship between WM and the ability to perceive sentences with degraded lexical tone information in the TTB may be a result of the effects of WM on top-down speech processing (i.e., *postdiction* based on the ELU model) [12]. That is, WM is deployed and used for making inferences and decisions to compensate for the mismatch. However, as discussed above, contextual information of the sentence lexical tones could help listeners unload WM, thus reducing the effects of WM in perceiving sentences with mismatched lexical tone information [11]. The current study cannot determine how the above three factors (i.e., *prediction*, *postdiction*, and contextual information) affect the relationship between WM and perceiving sentences with mismatched lexical tones. Sentences with high, low, and no contextual information could be included in future studies to elucidate its mechanisms.

Moreover, results showed that the lowest Cantonese sentence recognition scores were obtained in FT 60% instead of FT 100% in the TTB. Although the FT 60% condition provided more lexical tone information than the FT 100% condition, the former may have deployed more WM capacity than the latter. FT 100% sentences made it easier to “track” the target stream, thereby making sentence recognition less cognitively demanding, especially for attention and WM [26]. This finding may explain why some novel cochlear implant (CI) speech processing strategies designed explicitly to incorporate more tonal information have failed to contribute significantly to sentence recognition in noise compared to present CI speech processing strategies. More specifically, current vocoder-centric CI speech strategies may not provide sufficient spectral cues to encode the F0 cues, leading to poor lexical tone and sentence perception [1, 6]. Many novel CI speech processing strategies have attempted to improve sentence recognition by enhancing F0 information or temporal periodicity cues critical for tone perception (e.g., Milczynski, Chang, Wouters, & Van Wieringen [28]; Vandali et al. [29]). However, these processing strategies may introduce more distortions, thereby requiring more WM capacity. Consequently, sentence recognition may be a result of the trade-off effects among enhanced temporal and spectral cues, distortions introduced by these enhanced cues, and WM. CI users with poor WM may be more likely to benefit from CI speech processing strategies that are less cognitively taxing than those providing more lexical information but demanding more cognitive resources.

## Conclusions

This study altered both primary and secondary cues critical for lexical tone perception by flattening the original lexical tones (Cantonese high-level tone, Tone 1) using a TTS synthesizer. Results suggest that lexical tone information for everyday Cantonese sentence recognition is relatively redundant in quiet but important in noise for adults with NH. In addition, WM is utilized when there is a mismatch in lexical tone information at the sentence level in the TTB, but not in the SSN, suggesting that the ELU model is also applicable to tonal languages when lexical tones are mismatched. Implications of the current results were discussed, and future studies were proposed.

## Supporting information

S1 Data (XLSX)
